# Controversies and Misconceptions Related to Cerebrospinal Fluid Circulation: A Review of the Literature from the Historical Pioneers' Theories to Current Models

**DOI:** 10.1155/2018/2928378

**Published:** 2018-11-26

**Authors:** Giorgio Mantovani, Marta Menegatti, Alba Scerrati, Michele Alessandro Cavallo, Pasquale De Bonis

**Affiliations:** ^1^University of Ferrara School of Medicine, Via Ludovico Ariosto, 35-44121 Ferrara, Italy; ^2^Neurosurgery, Sant'Anna University Hospital Ferrara, Via Aldo Moro 8, Cona (FE), Italy

## Abstract

Models of cerebrospinal fluid (CSF) circulation have been mainly proposed in the last century: CSF goes from the ventricles to the subarachnoidal space (SAS), passing through the aqueduct and the foramen of Luschka and Magendie. Indeed, new models, involving the Virchow-Robin space (VRS) and the perivascular space (PVS), have been proposed. We critically reviewed the literature, in order to clarify the “classical” errors and to discuss the “new” models that are evolving currently. Conclusions of past experiments are often not justified, due to lack of reproducibility and methodological issues. On the other hand, investigation on the microanatomy of Virchow-Robin spaces (VRS) and several new experiments showed a potential pathway for a more complex CSF “circulation,” with chaotic and unpredictable flows. It seems reasonable to elaborate a new model of CSF physiology, including new findings and questioning old certainties. However, proved data are still missing and it is hazardous to come to final conclusions. More studies are needed.

## 1. Introduction

All of us studied that cerebrospinal fluid (CSF) is produced by choroid plexuses (CP) (80%) and by ependyma (20%) and then circulates from the ventricles to the subarachnoidal space (SAS), passing through the aqueduct and the foramina of Luschka and Magendie. CSF is then reabsorbed into the venous sinuses, thanks to the arachnoidal granulations of Pacchioni. This movement is meant a real one, with a bulk flow originated by a mass transfer of molecules in a preferential direction.

Although anatomical description and functioning of ventricles date back to ancient Greece and Leonardo da Vinci, the actual model of CSF physiology is based on a series of experiments carried out, mainly in the last century, by famous neurosurgeons or neurophysiologists like Dandy, Cushing, or Retzius.

Nowadays, evidences not coherent with the classical paradigm are emerging. Several authors have arisen doubts on all steps of CSF circulation: the historical experiments present several methodological issues, and many researchers recently failed in reproducing those results.

In this dissertation, we review the literature to clarify the “classical” errors and to discuss the “new” model that is evolving by now.

## 2. Review

### 2.1. The Historical Papers on CSF Circulation

In 1764 Cotugno et al. [1] recognized that a watery solution was present in the cerebral ventricles, contradicting the galenic assumption of the* spiritus animalis.*

In 1825 Magendie [2] understood the physiological importance of CSF.

**Figure 1:**
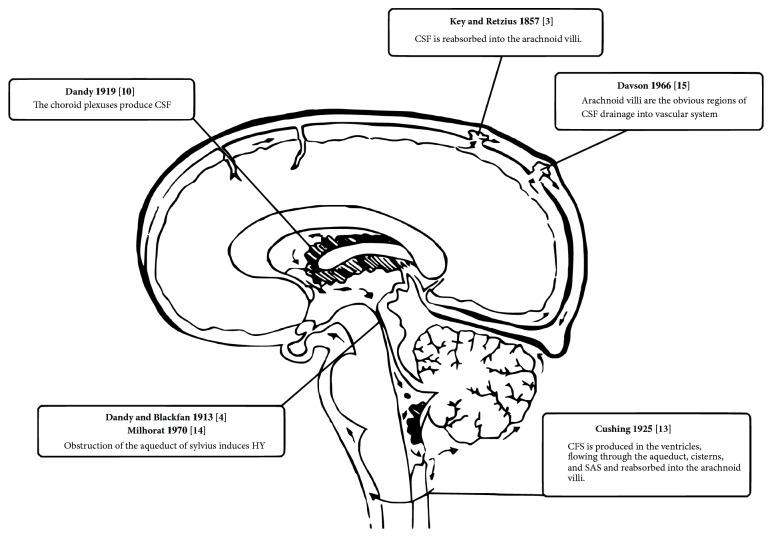
Evidences supporting the classic model of CSF circulation.

Experiments on CSF circulation that build our actual model of conceiving have been carried out over the past 150 years (Figure 1).

In 1875 Key and Retzius demonstrated that the CSF is absorbed by the arachnoid villi by injecting coloured gelatine into the SAS. The injection pressure was above 60 mmHg, causing the rupture of the villi with passage of gelatine in the lumen of venous sinuses [3].

In 1913, Dandy and Blackfan [4] induced hydrocephalus in dogs by obstructing the aqueduct of Silvius. In 1914, Frazier and Peet [5] and Wegefarth [6] were successful in reproducing Dandy's results, but in the meantime, many other authors [7, 8] failed. Furthermore, certain breeds of dog present occult ventricular enlargement or hydrocephalus in 60% to 80% of cases [9].

In 1914, Weed [3] repeated Key and Retzius experiment with a lower injection pressure (9-13mmHg), but he did not find any trace of coloured gelatine into the venous sinuses.

In 1919, Dandy [10] demonstrated on a single dog that the CP produces CSF, performing a bilateral obstruction of Monro foramina and a unilateral plexectomy. This determined the reduction of the operated ventricle and the dilation of the contralateral one. Again, the experiment could not be reproduced by others [7, 11].

Dandy introduced the choroid plexectomy as a treatment for hydrocephalus. The clinical results were so poor that in the 1950 most neurosurgeons abandoned this technique [12].

**Figure 2:**
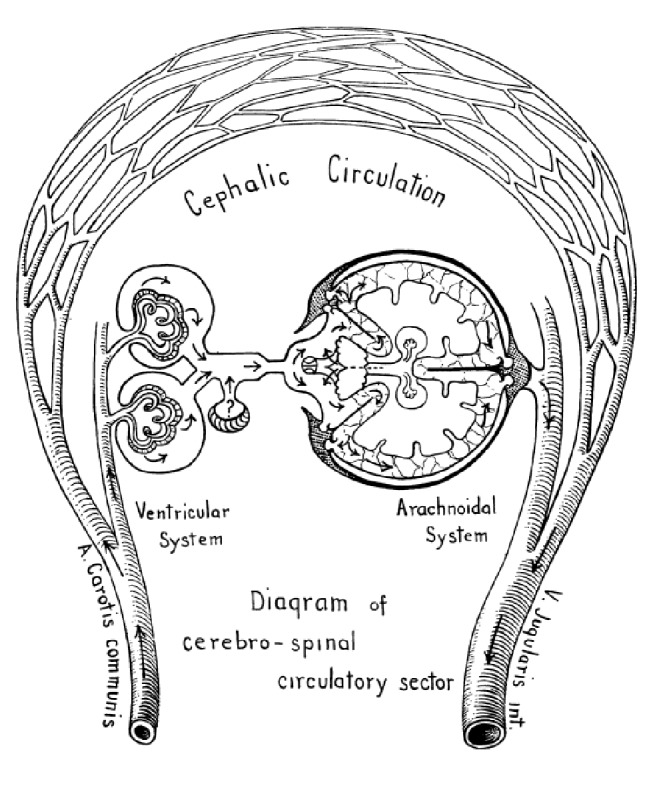
Cushing's model of CSF circulation [13].

In 1925, Cushing published “The third circulation and its channels” [13]. He introduced the model of CSF being produced in the ventricles, flowing through the aqueduct, cisterns, and SAS and being reabsorbed into the arachnoid villi (Figure 2). For the first time, he also suggested that CSF, like the lymph, “*proves to be in continual movement in a definite direction through a highly specialized pathway that cuts across the blood circle to envelop an organ in which a lymphatic apparatus of the usual type does not exist*”, serving as a vehicle for metabolites.

In 1970, Milhorat [14] reproduced Dandy's results about experimental hydrocephalus in monkeys. He entered the fourth ventricle through Magendie's foramen with n°8 Foley catheter. The catheter was then inflated, causing the sealing of the aqueduct. He reported a wide ventricular dilatation within few hours. The hydrocephalus was assessed both* in vivo* with ventriculography and* ex vivo* by analysing the specimen's brain.

In 1987, Davson [15] stated that arachnoid villi are* obvious* regions of CSF drainage into the vascular system,* “from a purely anatomic point of view”.*

### 2.2. Experiments Refuting the Historical Model of CSF Circulation

**Figure 3:**
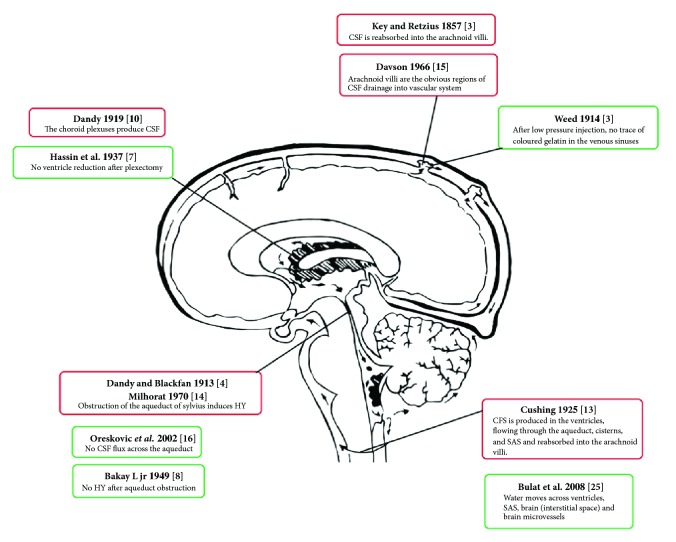
Evidences supporting (red) and confuting (green) the classic model of CSF circulation.

Over the last thirty years, the classical paradigm of CSF circulation has been questioned (Figure 3).

#### 2.2.1. CSF Production

Hassin et al. [7] in 1961 performed a monolateral plexectomy in dogs. No signs of reduction of the ventricles were observed. They concluded that a different source than the CP should exist.

Magnus [16] and Milhorat [17] stated that the fluid observed and collected on the surface of an isolated CP is not a valid representation of the CSF formation, because of the grossly unphysiologically state of a wide opened and drained ventricle.

#### 2.2.2. CSF Pathways and Connectivity to ISF

Oreskovic et al. [18] investigated CSF flow through the aqueduct in cats. They cannulated the aqueduct without stretching it and directly registered the CSF flow out from the third ventricle. They only observed a pulsation at the top of the cannula without any outflow. Hence, they injected artificial CSF from one lateral ventricle. In these conditions, a real outflow was observed.

They also injected a hyperosmolar solution of saccharose, observing an increased outflow. This proved that also osmolarity influences CSF physiology [19].

The common bias of exploring CSF circulation by injecting tracers in the SAS is that tracers' density is higher than CSF. Once injected, tracers would reach the lowest position pushed by gravitational force, i.e., the aqueduct.

For this reason, Bulat et al. [20] infused 3H-water (having the same colloidal features of normal water) into lateral ventricles or SAS of cats, measuring its absorption into cerebral microvessels.

**Figure 4:**
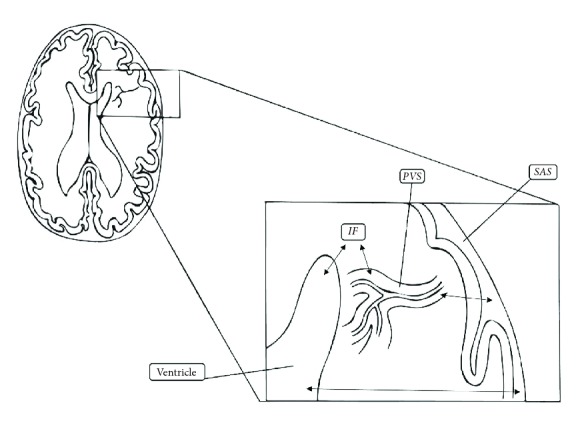
The “new” fluid balance model. IF: interstitial fluid; PVS: perivascular space; SAS: subarachnoid space.

During a three-hours infusion, the venous concentration of 3H water sharply increased, while arterial and CSF concentration remained stable. Their explanation consisted of a continuous turnover of water across microvascular walls, with a continuity of IF and CSF (Figure 4). 3H-water also passes from subarachnoid space across pia mater into cerebral microvessels. These finally drain into the sinuses.

The same procedure was performed with 3H-Inuline, which poorly passes across cerebral microvascular walls. 3H-inuline increased in CSF compartments and remained stable in arterial and venous compartments. They observed a bidirectional 3H-inuline distribution between cisternal CSF and lateral ventricle and between cisterna magna and cortical CSF. They assumed that substances slowly eliminated into microvessels spread not only along CSF spaces, but also between CSF and brain parenchyma. Subsequently they are slowly eliminated across capillary walls into bloodstream.

They concluded that neither the net formation of CSF volume in brain ventricles nor its unidirectional flow in subarachnoid space is present.

This is coherent with the observation that intrathecal infusion of drugs leads to a wide diffusion of the therapeutic molecules over the parenchyma [21].

In 1851 and in 1859, Virchow and Robin described the pia mater surrounding the vessels. Originally, it was thought that this space served as a communication to the subarachnoidal space (SAS) and as an alternative CSF draining towards the arachnoid villi [3].

Then, the Virchow-Robin space (VRS) was shown to be a virtual space, enlarged only in pathological processes [22].

Nowadays, the original idea about the function of this perivascular space (PVS) has been restored [23].

The PVS can be identified on larger cerebral blood vessels in both the subarachnoid spaces and within the parenchyma [24]. It is a fluid compartment within the outer walls of vessels, the basement membrane surrounding smooth muscle cells of tunica media and the adventitial connective tissue. It potentially extends downward, where tunica media becomes thinner and incomplete, and astroglial and capillary basal membrane fuse [24].

The first evidence of an intraparenchymal bulk flow along this PVS was provided by Cserr [25]. They recorded that Dextran Blu 2000, injected in the caudate nucleus of rats, was rapidly transported along extracellular and perivascular channels to the adjacent structures [25]. The observed flows were small and with unpredictable direction [26].

A consistent movement of fluid was also demonstrated by Rennels [27], by injecting horseradish-peroxidase into the lateral ventricle, or SAS of cats and dogs. They showed the tracer diffusion along the capillary pathway inside the parenchyma and they showed that horseradish-peroxidase influx could be prevented by diminishing the pulsation of cerebral arteries. This could suggest that hydrostatic forces drive water and solutes along the PVS, realizing an effective communication between CSF and interstitial fluid (IF).

In its complexity, the perivascular flow could consist of a sort “lymphatic system” [23]. The interstitial fluid (ISF) and the CSF could represent a homeostatic system; the idea of a “glymphatic” system for waste clearance from brain was coined by Iliff [28].

The Aquaporin-4 (AQP4) water channels are involved in this glia-mediated convective transport of fluids and solutes through the brain extracellular space [29].

Notably, AQP4 shows a polarized expression along CSF and parenchyma interface: pial surface-facing glia limitans, ventricle-facing ependymal cells, and perivascular end feet of astrocyte [29].

AQP4-null mice demonstrated an increased brain extracellular space volume fraction [30] and increased ICP in response to induced vasogenic edema [31]. Desai et al. [32] suggested that modulation of AQP4 could have a role in the treatment of hydrocephalus.

Many evidences question the proposed ‘glymphatic' system and the role of AQP4.

These molecules are not able to transport macromolecules [33] and there are no convincing evidences of a direct flow based on hydrostatic pressure/osmotic gradient, as it would be required to generate a convection from the arterial PVS to the venule PVS through the neuropil interstitium [34].

Aquaporins clearly have a central role in brain water balance, both in health and disease [35, 36], but what role astrocytic AQP4 may play in perivascular fluid circulation is yet unresolved.

## 3. Discussion

Our knowledge on CSF circulation is based on historical experiments. The major issue with those experiments is that all these studies involved rather drastic experimental procedures. A classical model of CSF circulation is no longer acceptable, since almost all its steps have been rebutted (Figure 2): CSF production (from CP to whole brain and vessels), CSF absorption (from Pacchioni granulations to brain and interstitial space), and CSF flow (from a unidirectional flow to an equilibrium among ventricles, parenchyma, vessels, and interstitial fluid).

CSF production in CP was demonstrated by Dandy with only one experiment. Hassin et al. [7], revealing the absence of ventricles reduction after plexectomy, concluded that the brain parenchyma was the main source of CSF production. Milhorat excluded that experimental hydrocephalus was secondary to fluid secreted by the choroidal plexus [12]. In fact, the animals who underwent plexectomy and aqueductal obstruction presented either hydrocephalus or normal ventricles, thus suggesting that another source of CSF production exists. The final demonstration of the inconsistency of this hypothesis was the failure of plexectomy in treating hydrocephalus [37]^.^

The belief that CSF is absorbed from the subarachnoid space to sagittal sinus through arachnoid villi arises from the experiments of Key and Retzius. Weed et al. [3] perfected these experiments by lowering the injection pressure of the tracer and found no gelatine in venous sinuses after infusion in SAS. Moreover, Bulat et al. showed that the tracer (tritiated water) passed from the brain to the veins, as CSF concentration of tritiated water did not vary during the experiment, while venous concentration continually increased [20].

The unidirectional CSF flow, described by Cushing, has evidences in the experiments of Dandy and Blackfan (1913) and Milhorat (1970). These authors obstructed the sylvian aqueduct of dogs (Dandy) and monkeys (Milhorat) and observed that animals developed hydrocephalus. Dandy experiment was very traumatic. Milhorat experiments (an inflated Foley) determined an intense parenchymal stretching of the aqueduct, causing a lumen dilatation more than twice [14].

Indeed, stenosis/obstruction of the sylvian aqueduct represents a known cause of hydrocephalus.

It is possible that aqueductal narrowing/closure may occur as a result and not as a cause of hydrocephalus. Experimental studies [38] have shown that mice infected by reovirus type-1 develop hydrocephalus. As the ventricles progressively dilate, midbrain compression results in aqueduct stenosis. This was confirmed in rabbits and mice [39, 40]. Foltz and Shurtleff [41] found that 12 among 27 patients with communicating hydrocephalus developed secondary aqueductal stenosis or aqueductal occlusion during chronic ventriculo-atrial shunting. Rados and Klarica [42] described a patient with aqueductal stenosis, due to a pineal cyst without hydrocephalus at 5-year follow-up. Some authors incorrectly state that cine-MRI provides evidence in favour of a CSF circulation through the sylvian aqueduct. This technique yields quantitative information about the aqueduct flow by synchronizing images acquisition with the cardiac cycle. The average flow is around 0.77 ml/min (ranging from 0 to 1.2 ml/min in different studies) [37] in the craniocaudal direction, but this value exceeds (more than double) the standard “choroidal production rate”.

Moreover, cine-MRI in children younger than 2 years old, or patients with hydrocephalus, often curiously reports an inverse flow, from the fourth to the third ventricle [43]. The algorithms used to obtain flow estimate in cine-MRI are based on the hypothesis that there is net craniocaudal CSF flow through the aqueduct, which diameter is assumed to be constant during a cardiac cycle (and this is hard to prove). In addition, the evaluation of flow void is highly subjective, also depending on acquisition parameters used [44].

The recent experiments, on the one hand, allow refuting the classical hypothesis, while on the other hand, they constitute the basis for a new model of “CSF noncirculation”. It would be more appropriate to talk about fluids movements, fluids exchanges among different intracranial compartments. These exchanges are represented by a dynamic equilibrium among parenchyma, interstitial space, SAS, vascular compartment, and ventricles (Figure 4). The hydrostatic and the osmotic forces seem to represent the main determinants of this dynamic equilibrium. In this perspective, the idea of the glymphatic has developed over the last years. However, recent evidences are discussing this hypothesis, in particular the role of glia and the transfer of solutes within the extracellular space [24].

If this model corresponds to what happens physiologically, several pathophysiological phenomena leading to pathological conditions must be reconsidered.

We could hypothesize the following phenomena:

- Hydrocephalus is not an imbalance between CSF production and resorption, but an imbalance between IF (decreased) and ventricular fluid (increased), while benign intracranial hypertension is an opposite imbalance (increased IF and decreased ventricular fluid).
- Hydrocephalus caused by tumoral aqueduct obstruction could be due to an increased osmolarity.
- Post-SAH hydrocephalus could be caused by an obstruction of IF and/or a hyperosmolar intraventricular/subarachnoid fluid.

This model could also provide a possible explanation for neurodegenerative disorders: could these pathologies have a correlation or a trigger in the interstitial fluid imbalance? In cerebral amyloid angiopathy, beta-amyloid is deposited around and in the vascular wall of arteries and arterioles. A dysfunction and obstruction of this drainage could be part of the aetiology of disease such as Alzheimer [45]. Indeed, interstitial fluid drainage is impaired in ischemic stroke and Alzheimer's disease mouse models [21, 46, 47].

More experiments are needed to better clarify the mechanisms determining the imbalance of fluids dynamic equilibrium in different pathological conditions.

## 4. Conclusions

The methods of historical papers, which have been used to determine the classical CSF circulation hypothesis, have very frequently been performed under nonphysiological conditions. The obtained results are therefore of questionable reliability.

New findings are emerging regarding the behaviour and the function of CSF.

The consequence of this conceptual evolution is that CSF physiology should not be considered as a unidirectional flow, through a unique anatomical well-defined pathway. More likely, it is a complex and dynamic equilibrium of fluids between vascular, neural, and cisternal spaces. In addition, the neurovascular interface is involved in the CSF production and resorption. This activity seems to be driven both by hydrostatic and osmotic forces, avoiding* de facto* the necessity of a real flow as classically described.

These considerations make the classic model of CSF circulation no longer acceptable.

## Abbreviations

SAS: Subarachnoidal space
BBB: Blood-Brain barrier
VRS: Virchow-Robin space
PVS: Perivascular space
IF: Interstitial fluid
CP: Choroidal plexus.
